# Treatment of exostosin 1-associated membranous lupus nephritis with multiple low doses of rituximab: A case report

**DOI:** 10.1097/MD.0000000000024887

**Published:** 2021-03-05

**Authors:** Ling Li, Zhi Yang, Tian Tao, Mei Yang, Zhang-Xue Hu

**Affiliations:** a Renal Division, Department of Medicine; b Department of Pathology, West China Hospital of Sichuan University, Chengdu, China.

**Keywords:** class V lupus nephritis, exostosin-1, membranous lupus nephritis, rituximab

## Abstract

**Rationale:**

Membranous glomerulonephritis (MN) is the leading cause of nephrotic syndrome in adults and is classified as primary or secondary. Secondary MN accounts for 20% to 30% of all MN cases and can arise from a number of conditions, including autoimmune diseases. Recently exostosin 1/exostosin 2 (EXT1/EXT2) have been identified as the common antigens in secondary autoimmune MN and are present in cases of pure membranous lupus nephritis (LN). The treatment of EXT1/EXT2-associated MN remains elusive.

**Patient concerns:**

We present the case of a 15-year-old female who presented with nephrotic syndrome, positive ANA and dsDNA, and low serum complements. A renal biopsy revealed pure membranous nephritis with IgG and C3 deposition. EXT1 was found along the glomerular capillary walls and stained positive, while phospholipase A2 receptor (PLA2R) and thrombospondin type-1 domain-containing 7A (THSD7A) were negative.

**Diagnosis:**

The patient was diagnosed with ETX1-associated membranous LN.

**Interventions:**

She was treated with prednisone and multiple low-dose rituximab (4 200 mg doses, approximately every 2 months, based on CD19+ cells counts).

**Outcomes:**

The patient had complete remission within 8 months later, and she remained in remission for the 16-month period of follow-up.

**Lessons:**

To our knowledge, this is the first case of EXT1-associated MN that has been successfully treated by multiple low-dose rituximab. Further studies can investigate the optimal dosage and treatment protocol.

## 1 Introduction

Membranous glomerulonephritis (MN) is the leading cause of nephrotic syndrome in adults. It is an organ-specific autoimmune disease, caused by the deposition of subepithelial immune complexes along the glomerular basement membrane (GBM). MN can be identified by light microscopy, which reveals thickening of the GBM; immunofluorescence microscopy, which shows granular staining for IgG and C3 along the glomerular capillary walls; and electron microscopy, which shows subepithelial GBM electron-dense deposits. MN can be classified as either primary or secondary, based on the identifiable causes.^[1]^ Primary MN is caused by antibodies directed against target antigens located on the glomerular podocyte; the primary target antigens are M-type phospholipase A2 receptor (PLA2R)^[2]^ and thrombospondin type-1 domain-containing 7A (THSD7A).^[3]^ Secondary MN can arise from conditions that include autoimmune diseases, malignancies, and infections.

The primary antigens for a subset of autoimmune diseases, including lupus, are exostosin 1/exostosin 2 (EXT1/EXT2).^[4]^ Exostosins are glycosyltransferases that are involved in the synthesis of complex polysaccharides,^[5,6]^ and they are the primary antigens for MN that is negative for PLA2R and THSD7A. The treatment for EXT1/EXT2-associated MN remains elusive, but rituximab (RTX) seems to be a promising option. RTX is a chimeric anti-CD20 monoclonal antibody that depletes CD20+ B cells, and it has been used to treat autoimmune diseases including vasculitis, immune hemolytic anemia, and rheumatoid arthritis. Recently, patients with membranous nephropathy at high risk for progressive disease experienced long-term proteinuria remission from treatment with RTX.^[7]^

We report on an EXT1-associated MN patient who went into complete remission by therapy with low-dose RTX and has remained in remission during a follow-up of 16 months. No other cases of EXT1/EXT2-associated MN treated with RTX have been reported to date.

## 2 Case description

A 15-year-old female was admitted to our hospital complaining of edema in the lower extremities, which had been occurring for the previous 2 months. She had no symptoms of fever, gross hematuria, foamy urine, photosensitivity, alopecia, oral ulcers, skin rash, joint pain, or urgency or pain in urination.

Her physical examination on admission showed no remarkable findings other than the mild edema of the lower extremities. Her body weight was 50 kg, height was 159 cm, and blood pressure was 114/82 mm Hg. There were no palpable lymph nodes, and the chest and abdominal exams were normal.

Laboratory tests showed hemoglobin, 129 g/L; white blood cells, 8.17 × 10^9^/L; and platelets, 151 × 10^9^/L. Urinalysis was positive for 4+ protein; RBC, 3/HP protein/creatinine ratio, 4.88 g/g Cr; and urine protein, 4.74 g/24 hour. Blood biochemistry analysis revealed albumin, 23.8 g/L; blood nitrogen urea, 5.60 mmol/L; serum creatinine, 50.0 μmol/L; uric acid, 208.0 μmol/L; and cholesterol, 6.24 mmol/L.

ANA was positive (1:1000); anti-dsDNA antibody was positive; anti-Sm antibody was negative; serum C3 was 0.6720 g/L (normal range 0.785–1.520 g/L); and C4 was 0.0806 g/L (normal range 0.145–0.360 g/L). There were negative findings for C-reactive protein, rheumatoid factor, anti-streptolysin O, anti-neutrophil cytoplasmic antibodies, and anti-glomerular antibodies. Immunofixation electrophoresis did not reveal monoclonal immunoglobulin. Renal ultrasonography showed that the size of the right kidney was 10.5 × 4.5 × 4.2 cm^3^, while the left measured 11.5 × 5.0 × 4.9 cm^3^.

**Figure 1:**
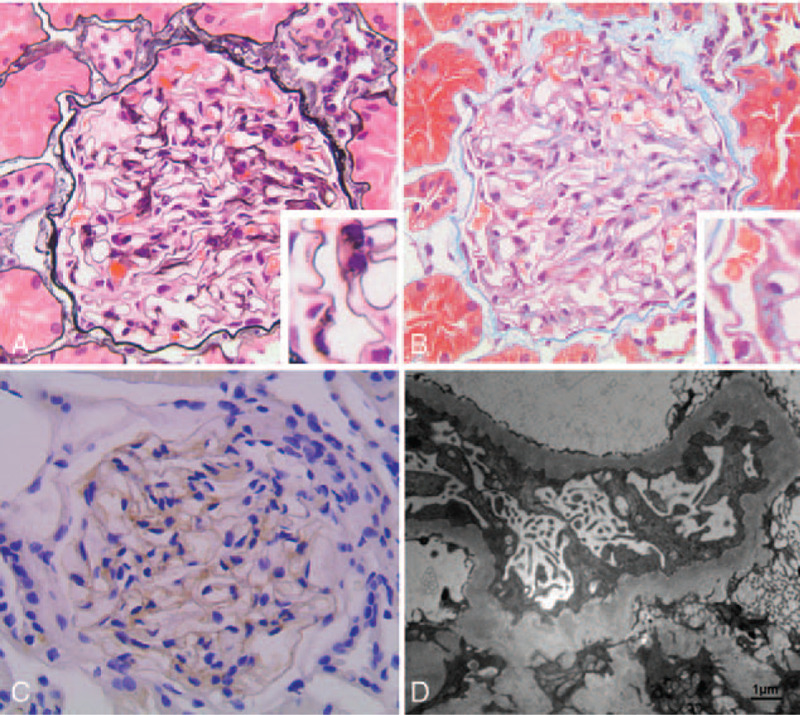
The histopathological findings of the renal specimen. (A) Light microscopy showed thickening of GBM (HE 400 ×). (B) Subepithelial fuchsinophilic protein deposition along the GBM (Masson 400 ×). (C) EXT1 staining was positive along the glomerular capillary wall (immunohistochemical staining, 400 ×). (D) Electron micrograph demonstrated subepithelial electron-dense deposits, thickening of GBM, and foot process fusion (scale bar=1 μm).

A renal biopsy was performed, and light microscopy revealed that 35 glomeruli had thickening of the GBM (Fig. 1A) and subepithelial fuchsinophilic protein deposition (Fig. 1B). Immunofluorescence microscopy revealed granular deposition of IgG and C3 along the glomerular capillary wall. The glomerular capillary was negative for PLA2R and THSD7A staining. An electron micrograph revealed subepithelial electron-dense deposits, thickening of the GBM, and foot process fusion (Fig. 1D).

We diagnosed the patient with membranous lupus nephritis (LN) (pure class V LN). We stained EXT1 by immunohistochemistry after the association of exostosin 1/exostosin 2 (EXT1/EXT2) and secondary MN was reported.^[4]^ The results showed positive staining for EXT1 along the glomerular capillary wall (Fig. 1C).

**Figure 2:**
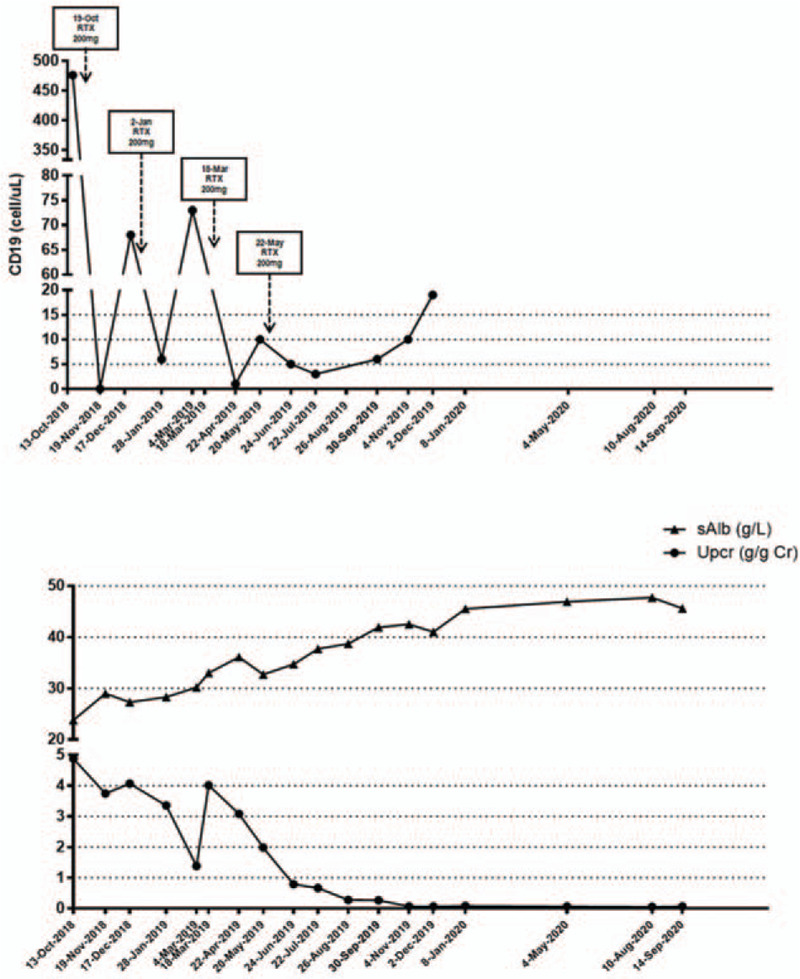
The clinical course of rituximab treatment. RTX = rituximab, sAlb = serum albumin, Upcr = urine protein/creatinine ratio.

The patient was given prednisone at a dose of 40 mg per day, which was tapered down after 1 month, hydroxychloroquine, and aspirin. Four 200 mg doses of RTX were given with the frequency of doses dependent on CD19+ B-cells counts; the average interval between doses was 2 months (Fig. 2).

The patient achieved complete remission 8 months after her hospital admittance. The serum albumin increased from 23.8 g/L to 38.7 g/L. The urinary protein creatinine ratio level dropped from 4.88 g/g Cr to 0.27 g/g Cr. The ANA and anti-dsDNA antibody were negative. The serum C3 increased to 0.7840 g/L. She remained in remission during a 16-month period of follow-up, with a maintenance dose of 7.5 mg of prednisone per day.

## 3 Discussion

Approximately 60% of patients with lupus have renal involvement, or LN, and 10% to 20% of these patients have membranous LN (class V), which can be pure or mixed with proliferative lesions of classes III and IV.^[8]^ Membranous LN, a secondary membranous nephropathy, is PLA2R and THSD7A negative. The antigens in secondary MN remained unknown until Sethi et al detected EXT1/EXT2 in both pure class V LN (8/18 patients) and in presumed primary MN associated with signs of autoimmunity (3/16 patients).^[4]^ These findings suggest that EXT1/EXT2 may represent the common target antigen of secondary (autoimmune) MN and could identify this special subset of LN.

Our patient was diagnosed with EXT1-associated membranous LN according to the Lupus International Collaborating Clinics classification criteria for systemic lupus erythematosus. She had EXT1-associated membranous nephropathy with nephrotic syndrome, high ANA titers, positive dsDNA, and decreased serum complements. A renal biopsy revealed EXT1-positive, PLA2R/THSD7A-negative, membranous nephropathy with IgG and C3 deposition.

RTX, a monoclonal antibody that specifically targets CD20 on the surface of B-cells, has been successfully used to treat lymphomas and some autoimmune diseases. B-cells play a central role in the pathogenetic process of LN. B-cell over-activity triggers the autoimmune processes associated with systemic lupus erythematosus, such as the production of autoantibodies and various cytokines, and it activates potent antigen-presenting cells.^[9]^

A recent treatment for LN is B-cell–targeted therapy, especially B-cell depletion. However, a 2012 study, the LUNAR trial, looked at the common LN treatment method of MMF plus corticosteroids and found no added benefit when RTX was added to the regimen.^[10]^ But a 2013 trial by Weidenbusch et al found that RTX induced some response (partial or complete) in 74% of patients with refractory LN.^[11]^ A multicenter retrospective study showed that rituximab alone 2 doses of 1 g every 15 days or 4 doses of 375 mg/m^2^ every week) was effective as a therapy for pure membranous LN.^[12]^

No further biomarkers have been identified to indicate the subset of LN that would have a positive response to RTX. In patients with idiopathic MN, RTX effectively achieved complete remission. The MENTOR study looked at RTX compared to cyclosporine and found RTX equal in inducing remission of proteinuria and superior in maintaining remission.^[7]^

The regimens for RTX treatment include those derived from the lymphoma protocol (4 weekly doses of 375 mg/m^2^), rheumatoid arthritis protocol (1 g every 15 days), or low-dose RTX (1 dose of 375 mg/m^2^ or 100–200 mg repeatedly based on the counts of CD19+ cells). Multiple low-dose RTX treatment was reported to be effective in treating idiopathic MN and had good safety and tolerability,^[13–15]^ RTX infusion dependent CD19+ cell counts. A low-dose RTX regimen could also greatly reduce the risk of infection and the economic burden of patients. For the patient refused to take immunosuppressive agent such as cyclophosphamide or calcineurin inhibitors, we gave our patient multiple low-doses of RTX (200 mg per dose, monitor the counts of CD19+ B cell monthly, repeated if the counts of CD19+ B cell >5 cell/ul accompany with the up-regulation of Upcr, totally 4 doses) plus prednisone. The usage of RTX is called low-dose RTX titration to deplete the B cell. She had a complete remission (normal sCr level if it was abnormal at baseline, or a sCr level of ≤115% of baseline if it was normal at baseline; and UPC ratio <0.5) that was maintained during a follow-up period of 16 months, and no adverse effects were observed.

Ours is the first case of EXT1-associated MN treated successfully with multiple low-dose RTX and suggests the efficacy of RTX on EXT1-associated MN with heavy proteinuria. The efficacy and optimal dosage of RTX in EXT1-associated MN should be investigated further as well as the question of whether RTX should be used in the maintenance period.

## 4 Conclusions

In conclusion, EXT1 has been identified as a primary antigen in secondary autoimmune MN, and RTX can effectively induce remission of EXT1-associated MN.

## Author contributions

**Conceptualization:** Zhang-Xue Hu.

**Data curation:** Tian Tao.

**Investigation:** Zhang-Xue Hu.

**Supervision:** Ling Li, Zhang-Xue Hu.

**Writing – original draft:** Ling Li, Zhi Yang, Mei Yang.

**Writing – review & editing:** Zhang-Xue Hu.
